# Anatomy of the Right and Left Ventricular Subvalvular Apparatus of the Horse (*Equus caballus*)

**DOI:** 10.3390/ani14172563

**Published:** 2024-09-03

**Authors:** Karolina Bielińska, Aleksander F. Butkiewicz, Hanna Ziemak, Maciej Zdun

**Affiliations:** 1 Department of Basic and Preclinical Sciences, Institute of Veterinary Medicine, Nicolaus Copernicus University in Torun, Lwowska 1, 87-100 Torun, Poland; 319365@stud.umk.pl; 2 Department of Veterinary Surgery, Institute of Veterinary Medicine, Nicolaus Copernicus University in Torun, Szosa Bydgoska 13, 87-100 Torun, Poland; ziemak@umk.pl; 3 Department of Animal Anatomy, Poznan University of Life Sciences, Wojska Polskiego 71C, 60-625 Poznan, Poland

**Keywords:** animal anatomy, heart, domestic horse, subvalvular apparatus, chordae tendineae

## Abstract

**Simple Summary:**

Understanding the precise anatomy of the left and right subvalvular apparatus in the domestic horse can contribute to the development of normal and comparative animal anatomy, as well as surgery, internal medicine, and biology. This study conducts such an analysis, taking into account the thickness of the left ventricular wall, the right ventricular wall, the interventricular septum, the ventricle’s length, and the entire heart’s width. Additionally, it examines the number of muscle bellies of the papillary muscles, the type of connection between the muscle and the heart wall, the height of muscle origin, and the length of the papillary muscle. This study calculates various proportions and compares them with descriptions of subvalvular apparatuses in other animal species, including humans, available in the literature.

**Abstract:**

Due to the growing interest among veterinarians and the increasing market demands, the development of equine cardiology is necessary. Currently, veterinary medicine for companion animals needs to catch up to human medicine—equine medicine included. A common condition in older horses is aortic valve regurgitation resulting from fibrosis, while its more severe form occurs in younger horses or develops due to a bacterial infection. Mitral valve regurgitation, especially dangerous due to the possibility of sudden death, has a better prognosis if the horse has valve prolapse. Tricuspid valve regurgitation usually does not pose a clinical problem, although its severe cases may lead to heart failure. Some pathologies can be treated surgically, which requires excellent knowledge of anatomy. The object of this study consisted of twenty domestic horse hearts. The focus was on the normal and comparative anatomy of the left and right subvalvular apparatus. The number of muscular bellies of the papillary muscles and the type of connection of the muscles were analysed. Moreover, the height of muscle originating from the ventricle wall was determined, the morphological regularity of the papillary muscle was assessed, and the chordae tendineae originating from the papillary muscles were examined. The conducted research allowed for comparing domestic horses with different species through other studies, the authors of which described this particular aspect. Interspecies similarities which may be correlated with the evolutionary relatedness, as well as differences that could reflect adaptation to different lifestyles, environmental conditions, or metabolic requirements of the animals, have been found. This study expands the knowledge of animals’ normal and comparative anatomy, and contributes to the development of veterinary surgery, internal medicine, and biology.

## 1. Introduction

The domestic horse (*Equus caballus*) is a relatively well-studied species, and heart diseases in these animals are known within the veterinary community. From a veterinary perspective, knowledge of anatomy as a fundamental science is crucial for understanding the physiology, and therefore the pathophysiology and pathological anatomy, of various conditions. Veterinarians frequently encounter different pathologies that, in addition to systemic disorders, have anatomical underpinnings resulting from tissue damage, among other factors. A thorough understanding of anatomy allows for a better grasp of the mechanisms behind these disorders and enables more effective planning of diagnostics and treatment.

Heart diseases in horses have long presented a challenge in veterinary medicine. Commonly encountered conditions include aortic valve regurgitation (*valva aortae*) [1], mitral valve regurgitation (*valva mitralis*) [2,3,4], and tricuspid valve regurgitation (*valva tricuspidalis*) [5].

Equine cardiology is a well-developed field of veterinary medicine. The increasing interest among veterinarians and the growing market demand create further opportunities for its continued development.

Currently, veterinary medicine for companion animals needs to catch up to human medicine, equine medicine included. Considering the determination and interest of the owners of horses in advanced and expensive treatments, there is hope for the development of cardiac surgery in these animals.

The right atrium (*atrium dextrum*) connects to the right ventricle (*ventriculus dexter*) through the right atrioventricular orifice (*ostium atrioventriculare dextrum*), through which flows deoxygenated blood. This orifice contains the right atrioventricular valve (*valva atrioventricularis dextra*), known as the tricuspid valve, composed of three cusps: the angular cusp (*cuspis angularis*), the parietal cusp (*cuspis parietalis*), and the septal cusp (*cuspis septalis*). There are papillary muscles (*mm. papillares*) on the right ventricle wall and interventricular septum, to which the tendinous chords are attached. Chordae tendineae are divided into true chordae, which connect the papillary muscle to the valve, and false chordae, which do not have a connection to the valve. True chordae are classified by authors as primary and secondary, with some authors also distinguishing tertiary chordae [6]. Primary chordae attach directly to the edge of the valve, secondary chordae connect to the ventricular surface of the valve leaflet, and tertiary chordae are attached to the base of the valve. The valve itself contains the rough zone, where the chordae tendineae attach, and the smooth zone, which lacks these attachments [7].

In the right ventricle, small papillary muscles (*mm. papillares parvi*) can be distinguished on the interventricular septum (*septum interventriculare*): the subarterial papillary muscle (*mm. papillaris subarteriosus*) below the opening of the pulmonary trunk, and the great papillary muscle (*m. papillaris magnus*) on the wall of the right ventricle. The wall of the right ventricle also features trabeculae carneae. Another valve through which blood flows is the pulmonary trunk valve (*valva trunci pulmonalis*), composed of three cusps: the right semilunar cusp (*valvula semilunaris dextra*), the left semilunar cusp (*valvula semilunaris sinistra*), and the intermediate semilunar cusp (*valvula semilunaris intermedia*).

Oxygenated blood passes through the left atrium (*atrium sinistrum*) to the left ventricle (*ventriculus sinister*), which is smaller in volume. In the left atrioventricular orifice (*ostium atrioventriculare sinistrum*), there is the left atrioventricular valve (*valva atrioventricularis sinistra*), known as the mitral valve (*valva mitralis*), composed of two cusps: the parietal cusp (*cuspis parietalis*) and the septal cusp (*cuspis septalis*). The left ventricle has two papillary muscles: the subauricular muscle (*m. papillaris subauricularis*) and the subatrial muscle (*m. papillaris subatrialis*). Similarly to the right ventricle, the presence of trabeculae carneae can be observed here [8].

In the widely available literature on the subject, publications have been appearing for many years [9,10,11]. Human anatomy studies dominate, though there are also numerous publications on animal anatomy [12]. It is worth noting that so far, no research has been conducted on the anatomy of the subvalvular apparatus in horses. The aim of this study was to determine the anatomical structure of the left and right subvalvular apparatus in the domestic horse. A detailed analysis of these structures can contribute to developing basic and clinical sciences in veterinary medicine. Knowledge of the detailed anatomy of the horse’s heart is fundamental for the further development of cardiology and cardiac surgery in veterinary medicine, enabling more accurate diagnosis and more effective treatment of heart diseases in these animals.

## 2. Materials and Methods

**Scheme 1:**
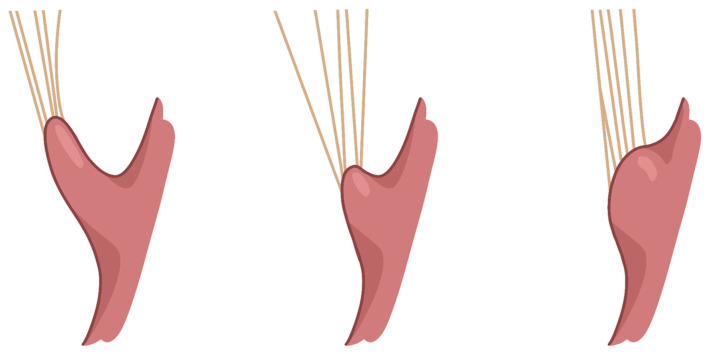
Types of connections of the papillary muscles. From the left side: 1—basal; 2—mixed; 3—intramural.

This study involved twenty hearts from healthy adult Wielkopolski horses without heart defects, sourced from the collection of the Veterinary Institute at Nicolaus Copernicus University in Toruń. The horses’ carcasses weighed between 430 kg and 560 kg. The animals ranged in age from 12 to 21 years. The hearts were preserved in a 5% formaldehyde solution. An ethical committee approval was not required, as the material was obtained post mortem. The hearts were stored in preserved form for two weeks. The right and left subvalvular apparatus were analyzed based on the methodology developed by Kustrzycki et al. [13]. Measurements (measuring to the nearest 0.01 cm using an electronic caliper) of the entire organ were taken, including the thickness of the left ventricular wall, the thickness of the right ventricular wall, the thickness of the interventricular septum, the length of the ventricle, and the width of the whole heart. Both sides of the heart were opened using a vertical incision running from the base of the heart to the apex along the middle of the free wall on each side. Measurements were taken at the widest, thickest, and longest sections of the organ. The measurement of the heart ventricle involved determining the distance from the apex of the heart to the coronary sulcus (*sulcus coronarius*). Each measurement was performed three times, and then the average of three measurements was calculated. This study also examined the number of muscular bellies of the papillary muscles, the type of connection between the muscle and the heart wall, the height of muscle origin, and the length of the papillary muscle. The length of the papillary muscle was measured from its base to the apex at the longest section using an electronic caliper while applying the aforementioned method of three measurements. Three types of connections were identified (Scheme 1):

- Basal type, in which the papillary muscle is fused with the ventricular wall only at the base.
- Mixed type, in which the papillary muscle partially adheres to the ventricular wall.
- Intramural type, where the papillary muscle is almost entirely connected to the ventricular wall.

**Figure 1:**
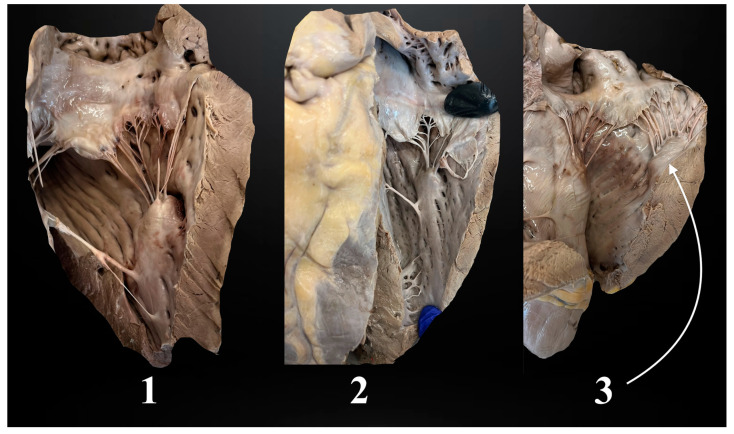
Classification according to the height of the papillary muscles (great muscle, right ventricle). 1—low; 2—medium; 3—high.

**Scheme 2:**
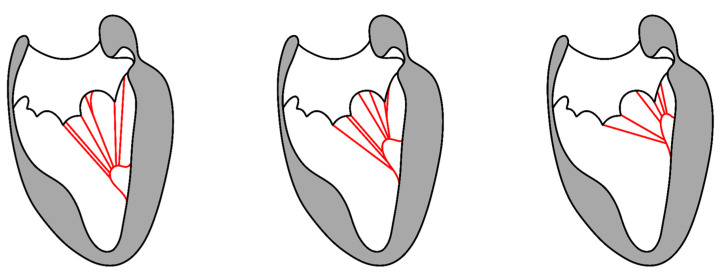
Classification types of connections of the papillary muscles. From the left side: 1—low; 2—medium; 3—high.

To determine the height of the papillary muscle origin, a line was drawn from the valve anulus (*anulus fibrosus*) to the apex of the heart (*apex cordis*), allowing classification as high, medium, or low (Figure 1 and Scheme 2). During the research, the chordae tendineae originating from the papillary muscles were also examined. The number of chordae and their attachment points to the rough zone and margin of the valve were determined, as well as the number of chordae tendineae extending from the papillary muscles to the corresponding valve leaflets. False chordae tendineae (*chordae tendineae spuriae*) were determined, as well. The length of the chordae tendineae was measured using the same methods, starting the measurement at the papillary muscle and ending it at the attachment to the valve. All of the measurements were taken by the same person for consistency.

Photos were taken with an iPhone 13 mini (Apple, Cupertino, CA, USA) and subsequently processed using the graphic software Preview Version 11.0 (1056.5.1), Copyright© 2002–2023 Apple Inc. (Apple, Cupertino, CA, USA). The nomenclature of the anatomical structures was standardized according to Nomina Anatomica Veterinaria (2017) [8]. Some of the terminology not found in Nomina Anatomica Veterinaria was borrowed from publications related to human medicine, including terms like “the rough zone of the valve”, “the smooth zone of the valve”, and “primary, secondary, and tertiary chordae tendineae” [5,6].

## 3. Results

This study showed that the minimal thickness of the interventricular septum was 3.75 cm, while its maximum was 5.93 cm. The mean septum thickness assumed a value of 4.48 cm. The average length of the ventricle was 19.22 cm, and the maximum and minimum values were 21.4 cm and 17.3 cm, respectively. The width of the heart of the studied species ranged from 15.60 cm to 19.90 cm. The average value equaled 17.91 cm.

### 3.1. Right Ventricle

**Figure 2:**
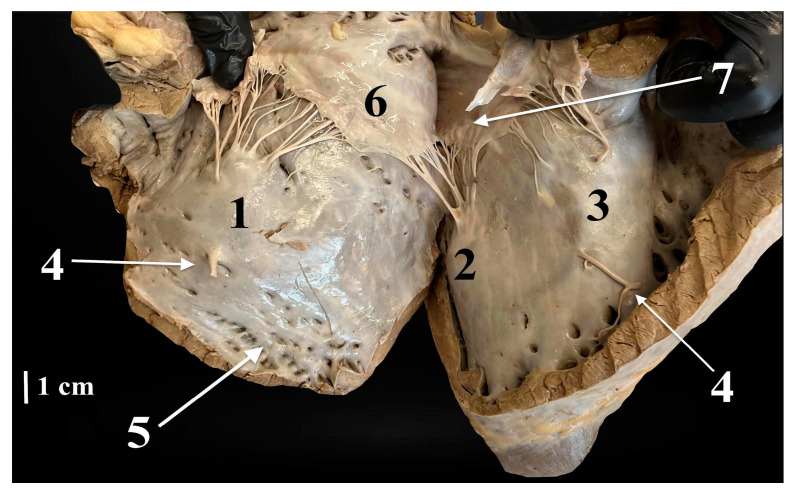
Right ventricle after cutting the angular cusp. 1—great papillary muscle (basal type); 2—subarterial papillary muscle (mixed type); 3—small papillary muscles (intramural type); 4—right septomarginal trabecula; 5—trabeculae carneae; 6—parietal cusp; 7—septal cusp.

**Figure 3:**
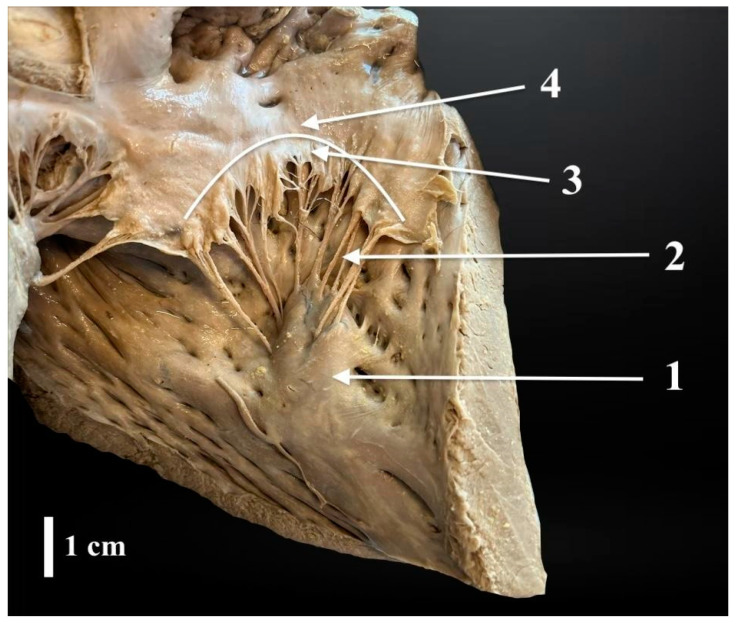
Right ventricle. 1—great papillary muscle (basal type connection); 2—tendinous chords; 3—rough zone of the tricuspid valve, parietal cusp; 4—smooth zone of the tricuspid valve, parietal cusp.

The right ventricle possessed papillary muscles: small, subarterial, and great (Figure 2). The small papillary muscles and subarterial papillary muscle originated from the interventricular septum, while the great papillary muscle was situated on the side of the right ventricle wall (Figure 3). The right atrioventricular orifice was equipped with a tricuspid valve composed of three cusps: the angulary cusp and the parietal cusp, attached to the free wall of the right ventricle, and the septal cusp, located on the right side of the interventricular septum. The mean length of the small papillary muscles was 2.73 cm. The minimum length in the case of these muscles was 1.22 cm, and the maximum length was 6.66 cm. In total, 80% of the papillary muscles had two bellies. For a single small papillary muscle, a presence of one head was observed. The remaining small papillary muscles were composed of three bellies. The length of the subarterial papillary muscles assumed an average value of 4.46 cm, while the minimum and the maximum length amounted to 2.74 cm and 8.42 cm, respectively. All subarterial papillary muscles were composed of only one belly. In the case of the great papillary muscles, the mean length was 4.78 cm. The minimum length was 2.51 cm, and the maximum length was 8.82 cm. The dominant type of construction, the same as in the case of the subarterial papillary muscles, was the single-bellied construction which occurred in seventeen hearts. The rest of the muscles were constructed with either two or three bellies. Interestingly, the studies showed that in the case of a heart with the longest small papillary muscles, the great papillary muscle also had the greatest length among the examined hearts. Similar relationships were not observed for muscles with the shortest lengths. The most common type of the small papillary muscle connection was the intramural type, and the least common one was the basal type. Mainly the mixed type was observed in the subarterial papillary muscle, appearing in 80% of the hearts, while 70% of the great papillary muscles were characterized by the presence of the basal type of connections (Table 1).

In the case of small papillary muscles, 50% of the muscles had high origin; however, the low origin of this muscle was observed in none of the examined hearts. In the subarterial and great papillary muscles, medium origin dominated, and high origin was noted in only three hearts (Table 2).

There were, on average, 26 tendinous chords in the right ventricle. In the examined hearts, an average of eight chordae tendineae branched from the small papillary muscles, with the majority of them reaching the septal cusp. The number of the chordae tendineae that extended from the small papillary muscles and headed towards the angle cusp and the septal cusp of the tricuspid valve totaled 32. The subarterial papillary muscles showed eight chordae tendineae, which reached the parietal cusp and the septal cusp in 41 branches, the majority of which reached the septal cusp. From the great papillary muscle, nine chordae tendineae were heading to the valve cusps—most of them reaching the parietal cusp, while the rest attached to the angulary cusp. The mean number of the branches of the chordae tendineae was, in this case, 46. The average length of the chordae tendineae in the right ventricle was 3.17 cm. In the right ventricle, the presence of the false chordae tendineae was confirmed to be an average of three, with the predominance of thin false chordae tendineae over the thick ones. Their maximum number was 10, which was observed in two hearts. Most frequently, they originated from the bellies of the great and subarterial papillary muscles.

### 3.2. Left Ventricle

**Figure 4:**
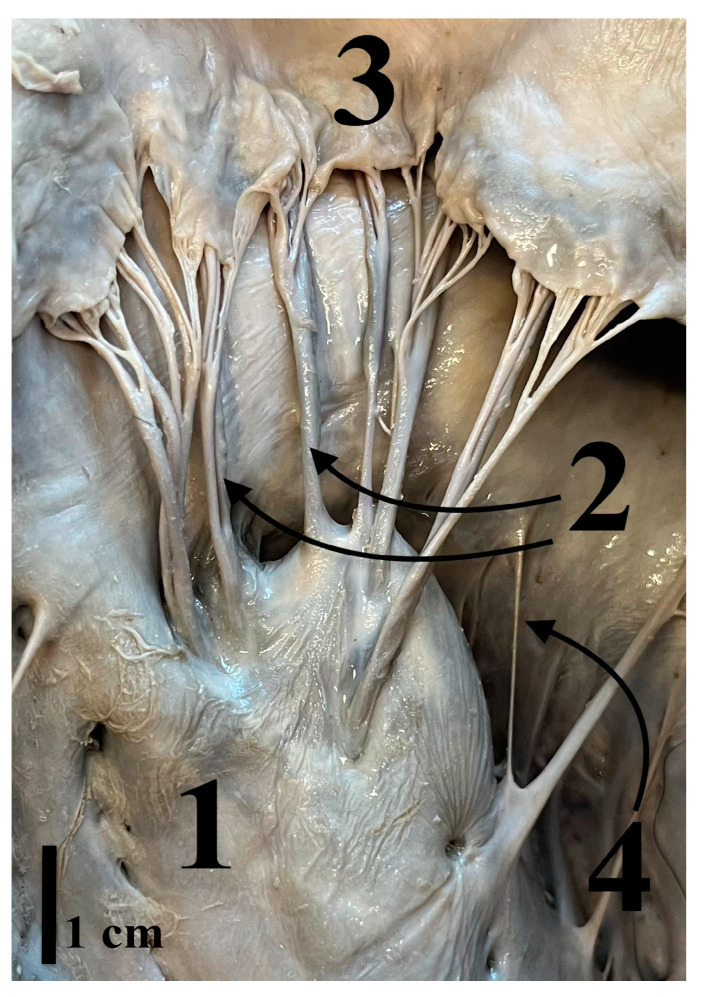
Basal type connection—papillary muscle (the image is captured from a close distance). 1—subatrial papillary muscle; 2—tendinous chords; 3—mitral valve; 4—false chord tendon.

**Figure 5:**
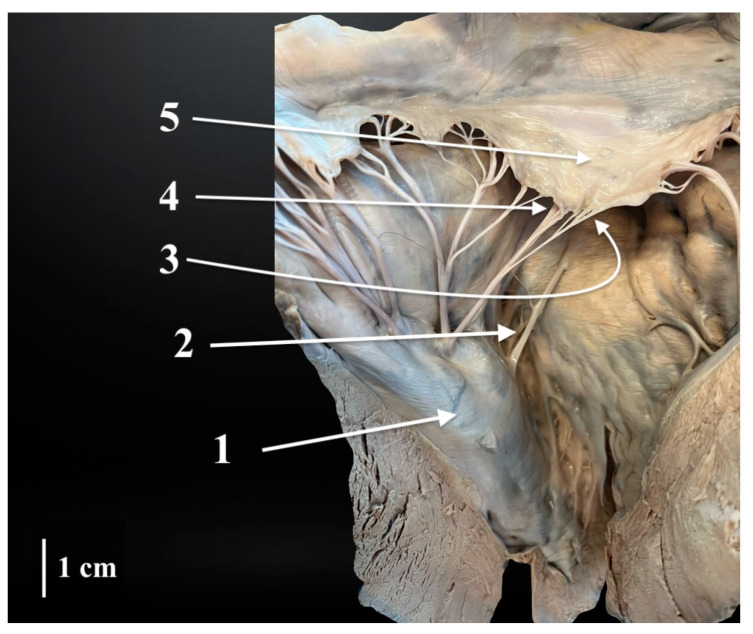
Left ventricle. 1—subauricular papillary muscle (mixed type connection); 2. false chord tendon; 3. primary chord tendon; 4. secondary chord tendon; 5. septal cusp.

The examination showed that the mean thickness of the left ventricle wall was 3.58 cm, while the minimum and maximum thickness assumed values of 2 cm and 4.64 cm, respectively. The left ventricle wall was equipped with two papillary muscles: the subatrial muscle (Figure 4) and the subauricular muscle (Figure 5). The chordae tendineae of these muscles headed towards the mitral valve located in the atrioventricular orifice. The valve was composed of two cusps: septal and parietal. The chordae tendineae were attached to the rough surface of each of them, providing the connection between the valve cusps and the papillary muscles. The length of the subauricular papillary muscle was, on average, 7.65 cm, while the minimum length was 4.23 cm and the maximum length was 11.1 cm. Most of these muscles were characterized by a presence of one belly; however, in the case of five examined hearts, the number of bellies equaled two. Only in three hearts were the muscles constructed with three bellies. Regarding the subatrial papillary muscles, the average length was 6.57 cm, the minimum length was 3.49 cm, and the maximum length was 9.82 cm. Like in the case of subauricular papillary muscles, the majority of the subatrial papillary muscles had only one head. The two-bellied type of construction was noted in the case of seven hearts. Moreover, the analysis also showed that in the case of a horse with the longest subauricular papillary muscle, the length of the subatrial papillary muscle was also the greatest among the examined horses. A similar relationship was not identified for the minimal sizes of the mentioned muscles, since in the case of a heart with the smallest subauricular muscle, the length of the subatrial papillary muscle was above average. The dominant type of connection between the subauricular papillary muscle and the left ventricle wall was the basal type, observed in 15 hearts. Regarding the subatrial papillary muscles, the basal type occurred in eleven hearts, while the mixed type was observed in nine hearts. The intramural type was not noted in any of the hearts (Table 3).

The prevalent quantity of papillary muscles of the left ventricle was characterized by low origin; while medium origin was found in 40% of the subauricular muscles and in 30% of the subatrial muscles. High origin was observed in none of them (Table 4).

The mean number of all chordae tendineae in the left ventricle was 13.6. In total, 6.8 chordae tendineae originated from each papillary muscle (Table 5).

There were, on average, 8.3 chordae tendineae reaching the parietal cusp, and 5.3 reaching the angulary cusp. The number of chordae tendineae branches reaching the valve cusp was, on average, 50 originating from the subatrial papillary muscle and 49 originating from the subauricular muscle. In the left ventricle, the mean length of the chordae tendineae was 4.78 cm. The same as in the case of the right ventricle, in the left ventricle, the presence of an average of three false chordae tendineae was observed, while their maximum was six. Most often, they originated from the bellies of the subauricular and subatrial papillary muscles, with most of them originating from the subatrial muscle.

It was shown that the ratio of the mean length of the right ventricular papillary muscle to the mean length of the ventricle took on the values 0.142:1, 0.249:1, and 0.232:1 for the small, great, and subatrial papillary muscles, respectively. In the case of the muscles of the left ventricle, the ratio took on the value of 0.398:1 for the subauricular papillary muscle and 0.342:1 for the subatrial muscle. The ratio between the lengths of the papillary muscles of each ventricle and the lengths of the tendon chords was calculated. It was shown that the ratio of the average length of the papillary muscles of the right ventricle to the average length of the tendon chords of the area was 0.861:1 for the small papillary muscles, 1.508:1 for the great papillary muscles, and 1.407:1 for the subarterial papillary muscles. In addition, for the left ventricular papillary muscles, the ratio took on the values of 1.600:1 and 1.375:1 for the subauricular and subatrial muscles.

## 4. Discussion

### 4.1. Right Ventricle

The location of the tricuspid valve is consistent with previous findings regarding the general heart anatomy of other species [14], as demonstrated through the conducted research. It consists of the angular, parietal, and septal leaflets, commonly described in humans as the anterior, posterior, and medial leaflets [15]. The leaflets are separated by commissures—the anteroposterior, anteroseptal, and posteroseptal—and are connected via chordae tendineae to the papillary muscles [16].

The papillary muscles of the right ventricle show interspecies variability in terms of their number. In most vertebrates, including the domestic horse, the right ventricle features small, subarterial, and great papillary muscles. In the pampas deer (*Ozotoceros bezoarticus*), three papillary muscles (subarterial, small, and great) were observed within the heart, attached to the chordae tendineae of the tricuspid valve. The great papillary muscle was located on the septal wall and connected to the subarterial papillary muscle through the right septomarginal trabecula (*trabecula septomarginalis dextra*) [17]. When describing human hearts, researchers usually use different nomenclature and describe the papillary muscles of the right ventricle as the anterior (equivalent to the great muscle), posterior, and septal papillary muscles, with the latter being highly variable in occurrence [15]. Similarly, in the hearts of domestic sheep (*Ovis aries*), domestic pig (*Sus domesticus*), and domestic cattle (*Bos taurus*) [18], the muscles are often named differently, as the parietal, septal, and angular muscles [19]. In our case, we decided to standardize the nomenclature according to Nomina Anatomica Veterinaria 2017 [8]. Interestingly, the case was different for the Ugandan giraffe (*Giraffa camelopardalis rothschildi*), where researchers did not observe the presence of small papillary muscles and described only the subarterial muscle, located on the septal wall, and the great muscle, attached to the septomarginal trabecula [20].

The available literature also allowed us to determine that the subarterial papillary muscle of the Ugandan giraffe was bilobed, while the great papillary muscle generally had a simple structure [20]. The small papillary muscles of the right ventricle of the domestic horse were characterized by the presence of two bellies in 80% of cases. Only one of these muscles had a single belly, while the remaining ones had three bellies. In all examined hearts of the domestic horse, unlike in the case of the Ugandan giraffe, the subarterial papillary muscles had only one head. The same was true for the great papillary muscles, which were also predominantly single-bellied. Research by Aktas et al. [21] on the papillary muscles of the right ventricle in human hearts showed that the anterior papillary muscles had a single or double belly structure, with the majority having a single belly. The posterior papillary muscles had between one and five bellies, with most having one or two bellies; while the septal papillary muscles had one to four bellies, with most having two bellies.

Regarding the type of connection between the papillary muscle and the ventricular wall, most species have yet to be thoroughly described in this case, suggesting the need for further research.

Regarding the height of the papillary muscle origin, the small papillary muscles of the domestic horse exhibited high origin in 50% of the examined hearts, and low origin was not identified in any of these muscles. Additionally, both the subarterial and great papillary muscles predominantly exhibited medium origin. In domestic cattle, researchers found that the parietal papillary muscles had origin at the level of the middle third of the right ventricle. In contrast, the septal and angular papillary muscles had origin at the level of the upper third of the right ventricle [19].

Another important element of the subvalvular apparatus is the chordae tendineae, the quantity of which varies between different species. Studies on the hearts of the domestic horse showed that the number of chordae originating from the small papillary muscles and connecting to the angular and septal leaflets of the tricuspid valve averaged 32. In contrast, the subarterial muscles had eight main chordae, which branched into 41 segments reaching the parietal and septal leaflets, with the majority reaching the septal leaflet. From the great muscles, nine main chordae tendineae extended towards the valve leaflets, with the majority reaching the parietal leaflet, and the rest reaching the angular leaflet. The average number of chordae branches in this case was 46. In domestic cattle, the average number of main chordae tendineae in the right ventricle was twenty-two, with an average of eleven reaching the parietal leaflet of the tricuspid valve, seven reaching the septal leaflet, and four reaching the angular leaflet [19]. In the domestic pig, the number of chordae tendineae in this area ranged from 23 to 26, with an average of 23.3, while in the domestic sheep, where researchers observed 18 to 28 chordae tendineae in the right ventricle, the average number was 22 [18]. In humans, the number of chordae tendineae was determined to be between three and eight for the anterior papillary muscle, with an average of 5.3. For the posterior papillary muscle, the number of chordae tendineae ranged from 1 to 6, with an average of 2.7, while the septal papillary muscle had between 1 and 8 chordae tendineae, with an average of 3.5 [22]. Differences in the average number of chordae tendineae in the tricuspid valve across different species are shown in Table 6.

It is also worth mentioning the false chordae tendineae of the right ventricle, which, although not typical components of the subvalvular apparatus, are also described in anatomical and histological studies of the heart, although much less frequently than in the case of the false chordae tendineae of the left ventricle [23]. Research by Ateş et al. [24] showed that the domestic pig has from two to six false chordae tendineae in the right ventricle. In the right ventricle of the domestic horse, the presence of these structures was also confirmed, with an average number of three, with a predominance of thin false chordae over thick ones. The maximum number of them in this species was ten, observed in two of the examined hearts. They most frequently originated under the bellies of the great muscles and from the bellies of the subarterial muscles. Researchers confirmed the presence of false chordae tendineae in human hearts and classified them into five types. Type I of the false chordae tendineae is located between the interventricular septum and the anterior papillary muscle; type II between the interventricular septum and the posterior papillary muscle; type III between the anterior leaflet of the tricuspid valve and the free wall of the right ventricle; type IV between the posterior papillary muscle and the free wall of the ventricle; type V between the anterior papillary muscle and the free wall of the ventricle [23].

### 4.2. Left Ventricle

The left atrioventricular orifice is equipped with the mitral valve, which consists of two leaflets: the parietal and the septal. In the human bicuspid valve, authors describe the presence of main leaflets: the larger anterior leaflet (*cuspis anterior*) and the smaller posterior leaflet (*cuspis posterior*), as well as small, complementary intermediate leaflets (*cuspis intermediae*) located in the angles between the main leaflets [15]. Under normal conditions, the leaflets come together during ventricular contraction, and the function of the subvalvular apparatus prevents the leaflets from moving into the left atrium [25]. The atrial surface of the normal mitral valve is smooth and forms the floor of the left atrium during ventricular contraction, while the ventricular surface is slightly irregular due to the attachment of the chordae tendineae [26].

Furthermore, the papillary muscles also play a significant role in the functioning of the valve, pulling the annulus of the atrioventricular valve towards the apex of the heart, and preventing excessive dilation of the ventricle [22]. As in the case of the domestic horse, the Eurasian wild boar (*Sus scrofa*) [27], the landrace pig [28], the African lion (*Panthera leo*) [29], the alpaca (*Vicugna pacos*) [30], the South American fur seal (*Arctocephalus australis*) [31], and the Ugandan giraffe [20], the left ventricle has two papillary muscles: the subauricular and the subatrial. In domestic cattle, the observed papillary muscles were described as the septal and parietal muscles [19], or alternatively as the anterior and posterior muscles [18]. The presence of the anterior and posterior papillary muscles was also observed in humans [15] and domestic sheep [18]. In addition, the left ventricle of the western grey kangaroo (*Macropus fuliginosus*) showed extensive development of two papillary muscles located on the parietal wall [32]. In the class Aves (birds), the common ostrich (*Struthio camelus*) exemplifies as having more than two papillary muscles in the left ventricle. Specifically, this species has three papillary muscles in its left ventricle: two situated on the lateral wall and one connected to the septum of the cardiac muscle [33].

The number of bellies of the left ventricular papillary muscles also varies by species. As mentioned, the majority of the subauricular muscles of the domestic horse was characterized by the presence of a single belly. However, in five of the studied hearts, there were two bellies. Only three of the hearts had subauricular papillary muscles consisting of three bellies. Similarly, the majority of subatrial papillary muscles in this species had a single belly. In contrast, the Eurasian wild boar predominantly has three-headed subauricular papillary muscles, occurring in 46.5% of examined hearts, while single- and two-headed types were found in 14.1% and 39.4% of hearts, respectively. In the case of the subatrial muscles of the wild boar heart, the three-bellied structure again predominated, while the one- and two-bellied types were present in 7% and 43.7% of the muscles studied, respectively [27]. The research showed that 96.7% of Landrace pig subauricular papillary muscles have a single head, while the remaining 3.3% have two heads. The subatrial muscles of this species mostly had a single head. Two bellies were present in 6.7% of the muscles, while three bellies were present in 3.3%. [28]. In human hearts, 82.5% of anterior (anterolateral) papillary muscles had a single head, with two or three heads observed in 14.3% and 2.9%, respectively. For posterior (posteromedial) papillary muscles, the dominant type was the double-headed variety, occurring in 54.3% of hearts. In comparison, single and three-headed types were found in 31% and 12% of the 105 examined hearts, respectively. Additionally, there were cases with more than three bellies [26]. Berdajs et al. [34] proposed the identification of papillary muscles by dividing them into several groups. In group I, the basal part and the apex of the muscle were undivided. In group II, the muscle had two heads; in subgroup II/A, the base of the papillary muscle was undivided, while in II/B, it was divided into two separate parts. In group III, the papillary muscle had three heads. In subgroup III/A, the base was undivided, while in III/B, it was made up of two, and in III/C, three separate parts.

The dominant type of attachment of the subauricular papillary muscle to the ventricular wall in domestic horses was the basal type. For subatrial papillary muscles, the basal type was present in 55% of hearts, while the mixed type occurred in 45% of the hearts. No intramural type attachments were observed in any of the examined hearts of this species. In Eurasian wild boars, the basal attachment type of subauricular papillary muscles was found in 30 of 71 hearts, mixed type in 29 hearts, and intramural type in 12 hearts. The subatrial papillary muscles predominantly had basal attachments, with mixed and intramural types occurring in 22 and 20 out of 71 examined hearts, respectively [27].

Regarding the height of muscle origin, the majority of left ventricular papillary muscles in domestic horses had low origin, with no cases of high origin reported. Medium origin was observed in 40% of subauricular papillary muscles and 30% of subatrial papillary muscles. In the Eurasian wild boar, the predominant part of the subatrial muscles was classified as having medium origin; high and low origins were present in 12.7% and 4.2% of the hearts, respectively. The subauricular papillary muscles had high origin in 57.7% of the cases and medium origin in 42.3% of the cases, with no cases of low origin [27]. In domestic cattle, each left ventricle muscle had its origin in the upper third of the ventricle [19]. A study by Agata Krawczyk-Ożóg et al. [5] measured the height of attachment of specific papillary muscles assigned to two groups, the superolateral and inferoseptal papillary muscle groups, to the left ventricular wall. This study showed that most muscles in the superolateral group were attached to the left ventricular wall at an average height of 20.9 mm (standard deviation 6.1 mm), while in the inferoseptal group, most muscles were attached at an average height of 18.6 mm (standard deviation 5.4 mm).

Another component of the left subvalvular apparatus are the chordae tendineae, which connect the atrioventricular valve leaflets to the papillary muscles in the heart ventricles. They prevent the valve leaflets from prolapsing into the atria during ventricular contraction, ensuring proper valve closure and preventing blood reflux. The average number of chordae tendineae in the mitral valve was 12 in domestic sheep, with a minimum of 11 and a maximum of 20. In domestic pigs and cattle, the average number of chordae was 16 and 20, respectively [18]. In domestic horses, the average number of chordae tendineae in the left ventricle was 13.6, with an average of 6.8 chordae from each muscle. An analysis of Eurasian wild boar hearts by Butkiewicz et al. [27] revealed that the number of chordae tendineae originating from the subauricular papillary muscle ranged from one to fifteen (average 6.45), with nine being the most common number found in 21.1% of the examined individuals. In the case of the subatrial papillary muscle, the number ranged from two to fifteen, with an average of nine. The average number of chordae tendineae from the subatrial papillary muscle to the parietal leaflet of the mitral valve was 5.15, and to the septal leaflet—2.92. For the subauricular papillary muscle, the average number of chordae to the parietal leaflet was 4.76, and to the septal leaflet it was 3.35. Similarly, in domestic horses, the total number of chordae to the parietal leaflet was an average of eight, and to the angular leaflet it was six. Interestingly, Ozbag et al. [35] observed that in domestic dogs (*Canis familiaris*), the number of chordae tendineae from the subauricular papillary muscle ranged from five to ten, and from the subatrial papillary muscle from five to thirteen. In domestic goats (*Capra hircus*), the number ranged from 4 to 10 for the subauricular papillary muscle, and from three to eleven for the subatrial papillary muscle.

Studies conducted on the human heart show that the number of chordae tendineae originating from the anterior and posterior papillary muscles is higher than in other examined mammals, ranging from six to twenty-one and from three to twenty-two, respectively [36]. Just as importantly, the total number of chordal branches reaching the rough surface of the valve leaflets in the Eurasian wild boar ranged from 100 to 250, with 63.4% of hearts containing between 130 and 190 chords, and the largest group (18.3% of the total number) having 150–160 chords [27]. In the domestic horse, these numbers were generally lower, with the number of chordal branches reaching the valves ranging from 69 to 179. The number of chordal branches reaching the valve leaflets from the subatrial muscle averaged 50, and from the subauricular muscle the number averaged 49. In the case of chordae tendineae rupture, flail valve movement is observed, with the leaflet falling into the left atrium and associated severe mitral regurgitation [25]. Table 7 presents the differences in the average number of chordae tendineae in the mitral valve among various species.

Additionally, studies often mention false chordae tendineae, which are chord-like structures that traverse the left ventricular cavity. They attach to the interventricular septum, papillary muscles, or free wall of the ventricle but are not associated with the mitral valve. They are present in approximately half of the human hearts examined during autopsies [37]. False chordae tendineae of various sizes and locations were detected in 77.9% of examined infants and children and in 61.8% of cases with congenital heart defects. They were also present in 95.0% of piglet hearts [38]. In the Eurasian wild boar, all examined hearts had false chordae tendineae in the left ventricle, usually in numbers ranging from one to two [27]. In the domestic horse, the average number of false chordae tendineae in the left ventricle was three, with only one examined heart lacking their presence.

## 5. Conclusions

The conducted studies have shown that the subvalvular apparatus of a horse is characterized by a structure typical for many mammalian species, with no significant morphological differences observed in comparison with most of the studied species. It has been demonstrated that the average size of the papillary muscles in the left ventricle of the heart was larger than that in the right ventricle, which may result from the different functions performed by various areas of the heart. The predominant muscle structure in most of the heart muscles of the domestic horse was found to be single-bellied. The analysis of the left ventricle of the domestic horse’s heart also revealed that most of the papillary muscles in this area are characterized by the presence of basal-type connections, which again indicates similarities between the compared animal species. In terms of the height at which the muscles originate from the wall of the left ventricle, the majority of the muscles were characterized by a low origin, which was not typical for the species with which the horse was compared in the study. However, in the case of the right ventricle, the basal connection was dominant in the great papillary muscle, while the small and subarterial muscles featured mixed and intramural types, respectively. The average number of all chordae tendineae in the left ventricle was 13.6, with 6.8 tendons from each papillary muscle, showing similarity to domestic sheep, domestic goat, domestic pig, and Eurasian wild boar. In the right ventricle, statistically, more chordae tendineae originated from the great muscle than from the small and subarterial muscles. The horse has a similar number of chordae tendineae in the right ventricle as domestic cattle, domestic sheep, and domestic pig. Furthermore, false chordae tendineae were observed in both the right and the left ventricle, with an average number of three.

## Figures and Tables

**Table 1:** Type of connection between the muscles and the heart wall in the right ventricle.

Type of Connection	Small Muscle		Great Muscle		Subarterial Muscle	
	Number of Hearts	%	Number of Hearts	%	Number of Hearts	%
	N = 20		N = 20		N = 20	
Basal type	2	10	14	70	2	10
Mixed type	3	15	4	20	16	80
Intramural type	15	75	2	10	2	10

**Table 2:** The height of muscles origin in the right ventricle.

Height of Muscle Origin	Small Muscle		Great Muscle		Subarterial Muscle	
	Number of Hearts	%	Number of Hearts	%	Number of Hearts	%
	N = 20		N = 20		N = 20	
High	10	50	3	15	3	15
Medium	10	50	13	65	12	60
Low	-	-	4	20	5	25

**Table 3:** Type of connection between the muscle and the heart wall in the left ventricle.

Type of Connection	Subatrial Muscle		Subauricular Muscle	
	Number of Hearts N = 20	%	Number of Hearts N = 20	%
Basal type	11	55	15	75
Mixed type	9	45	5	25
Intramural type	-	0	-	0

**Table 4:** The height of muscles origin in the left ventricle.

Height of Muscle Origin	Subatrial Muscle		Subauricular Muscle	
	Number of Hearts N = 20	%	Number of Hearts N = 20	%
High	-	0	-	0
Medium	6	30	8	40
Low	14	70	12	60

**Table 5:** Tendinous chords extending from the papillary muscles of the left ventricle.

Number of Tendinous Chords	Subatrial Muscle		Subauricular Muscle	
	Number of Hearts	%	Number of Hearts	%
	N = 20		N = 20	
3	1	5	-	0
4	2	10	1	5
5	2	10	2	10
6	4	20	6	30
7	3	15	5	25
8	3	15	4	20
9	4	20	1	5
10	1	5	1	5

**Table 6:** Average number of chordae tendineae in the tricuspid valve across different species, including both primary and secondary chordae.

Species	Average Number of Chordae Tendineae
Domestic horse	26
Domestic pig	23.3
Domestic cattle	22
Domestic sheep	22
Human	11.5

**Table 7:** Average number of chordae tendineae in the mitral valve across different species, including both primary and secondary chordae.

Species	Average Number of Chordae Tendineae
Domestic horse	13.6
Domestic pig	16
Eurasian wild boar	15.45
Domestic cattle	20
Domestic sheep	12

## Data Availability

The data presented in this study are available on request from the corresponding author.
